# *Plasmodium* species infecting lizards in the Brazilian Cerrado: Identification and description of a novel species, *Plasmodium brasiliensis* n. sp.

**DOI:** 10.1371/journal.pone.0319402

**Published:** 2025-04-02

**Authors:** Izabelle T. S. Carvalho, Adriana P. Furtado, Matheus A. Duarte, Pedro H. O. Pereira, Lívia S. M. Paiva, Júlio M. A. Silva, Guarino G. Colli, Erika M. Braga, Giane R. Paludo

**Affiliations:** 1 Laboratory of Veterinary Clinical Pathology and Molecular Diagnostics, College of Agronomy and Veterinary Medicine, University of Brasilia, Brasília, Distrito Federal, Brazil; 2 Veterinary Clinical Sciences, Washington State University, Pullman, Washington, United States of America; 3 Department of Parasitology, Universidade Federal de Minas Gerais, Brazil; 4 Department of Zoology, University of Brasília, Brasília, Distrito Federal, Brazil; Universidade Federal de Minas Gerais, BRAZIL

## Abstract

Parasites of the genus *Plasmodium*, responsible for lizard malaria, are widely distributed and taxonomically diverse. Molecular techniques provide valuable insights into the evolutionary relationships of *Plasmodium* species and lineages. However, the available data are still scarce, emphasizing the need for taxonomy-focused studies. We investigated the occurrence of *Plasmodium* infection in free-living lizards in the Brazilian Cerrado using an integrative approach based on the amplification/sequencing of the *Plasmodium cytb* gene and microscopic analysis. Among 302 lizards screened, 61 (20.2%) tested positive in the molecular screening, including 18 with concordant results in microscopy. We recovered 16 sequences in the first molecular survey of this host group in the biome. Our findings unveil a variety of *Plasmodium* species, some of which were previously undocumented in this region. We describe new lineages of *Plasmodium ouropretensis*, *P. tropiduri*, *P. carmelinoi*, and also report a new species, herein named as *Plasmodium brasiliensis* n. sp. The infection by *Plasmodium* sp. in *Hoplocercus spinosus* represents the first description of Apicomplexa organisms in this species. Furthermore, our results open remarkable possibilities for extensive studies in a field unexplored for many years.

## 1. Introduction

*Plasmodium* spp., or malaria parasites, comprise protozoans belonging to the phylum Apicomplexa, family Plasmodiidae. They exhibit vast taxonomic diversity and have a global distribution across all tropical continents and temperate areas. These intracellular protozoan parasites exploit a wide range of invertebrate and vertebrate hosts, including mammals, reptiles, and amphibians [1–3].

Historically, before the advent of molecular biology, parasitologists relied on optical microscopy, host switching, life history features, vectors, and habitat preferences for taxonomic classification. However, with the incorporation of molecular testing, the accuracy of these classifications has come into question [2,4,5]. Morphological traits observed in light microscopy are often distorted by preservation methods, and life history traits can evolve convergently in response to ecological pressures experienced by parasites [2]. Moreover, molecular studies have revealed independent evolutionary histories for morphologically similar parasites [6–8], emphasizing the importance of an integrated molecular approach.

Species delimitation for Haemosporida remains controversial within the scientific community [8,9]. For instance, Hellgren et al. [10] and Valkiūnas et al. [11] suggested that *cytb* lineages in *Haemoproteus* with genetic distances greater than 5% represent different species. However, in the same study by Hellgren et al. [10], two morphospecies differed by only 0.7%. A genetic distance of 3% in *Plasmodium* in lizards may indicate different species, even with high morphological similarities [6,9]. Average variations in genetic distance between *cytb* sequences for *Plasmodium* species in reptiles range from 1.7% to 4.8% [8], highlighting the importance of employing genetic, microscopic, and ecological tools simultaneously for accurate taxonomic classification.

Investigations involving *Plasmodium* in lizards with associated molecular data are scarce in Brazil [3,8,12]. Picelli and co-workers [13] presented a morphological survey of hemoparasites in lizards in the Brazilian Amazon region, where the order Haemosporida had a higher prevalence of infected animals, indicating the apparent spread of this agent in this biome. No reports with molecular data on *Plasmodium* in lizards in the Cerrado exist, and morphological data on these parasites are limited and sporadic [14,15].

The Brazilian Cerrado, home to numerous endemic species, is recognized as a global biodiversity hotspot [16], currently hosting 74 known lizard species, 45% of which are endemic [17]. Despite its ecological significance, the biome faces longstanding challenges, including limited protection of natural areas and a lack of studies on its rich biodiversity, particularly microorganisms, fungi, and invertebrates [16]. Here, we investigate the occurrence of *Plasmodium* in free-living lizards from the Cerrado, employing a combination of molecular techniques and microscopy in the first molecular survey of this parasite group in the biome.

## 2. Materials and methods

### 2.1 Sampling locations

From January 2018 to November 2020, we sampled lizards in different regions of the Brazilian Cerrado: IBGE Ecological Reserve (15°56’41” S and 47°53’07” W), Brasília, Federal District; Serra das Confusões National Park (9°28’1.11” S, 44°25’20.31” W; 44°21’33.91” W, 9°25’20.17” S, 44°19’56.14” W, 9°28’26.74” S, 44°18’27.14” W, 9°27’47.49” S), Redenção do Gurguéia, Piauí (Brazilian state); and Parque Municipal do Bacaba (14°42”23.88” S, 52°21’9.59” W), Nova Xavantina, Mato Grosso (Brazilian state). Using pitfall traps with drift fences [18], we captured 302 free-living lizards. We collected blood samples always in the morning, from the ventral caudal or jugular veins, respecting the limit of up to 1% of body mass, using 25x7 or 20x5 gauge needles, depending on the animal’s size, and 1ml syringes pre-rinsed with EDTA to prevent sample coagulation. In the field, we prepared thin blood smears in duplicate on glass slides specific for microscopy, using about 5 μl of the initial blood samples. We air-dried smears at room temperature and stored them for later staining.

In the field, we stored blood samples in a refrigerated thermal box. In the field laboratory, we centrifuged samples to separate plasma and cell concentrate and froze them. In the Veterinary Clinical Pathology Laboratory of the University of Brasília, we subjected the cell concentrates and blood smears to molecular testing and microscopic analysis, respectively. Our study was approved by the Ethics Committee for Animal Use (CEUA) of the University of Brasília under number 43/2019 and by the Biodiversity Authorization and Information System (SISBIO), number 62568-2.

### 2.2 Nomenclatural acts

The electronic edition of this article complies with the requirements of the amended International Code of Zoological Nomenclature; therefore, the new names contained herein are available under that Code from this electronic edition. This published work and the nomenclatural acts it contains have been registered in ZooBank, the online registration system for the ICZN. The ZooBank LSIDs (Life Science Identifiers) can be resolved and the associated information viewed through any standard web browser by appending the LSID to the prefix “http://zoobank.org/”. The LSID for this publication is: urn:lsid:zoobank.org:pub: 26592D81-D9F4-4BB3-88D1-02CFDB381AD9. The electronic edition of this work was published in a journal with an ISSN, and has been archived and is available from the following digital repositories: PubMed Central, repositorio.unb.br.

### 2.3 Microscopic analysis

Using the Rosenfeld method, we stained blood smears and mounted slides with Entelan and coverslips to preserve the material’s long-term quality. We analyzed the blood smears using an Olympus CX31 microscope. Digital images were captured using an Olympus Qcolor 5 camera and processed with QCapture software. Measurements were taken digitally using ImageJ 1.8.0 [19] and the morphometric parameters were obtained following the descriptions provided by Valkiunas [20].

### 2.4 DNA Extraction and polymerase chain reaction (PCR)

Following the manufacturer’s protocol, we extracted DNA from 10 μl of blood samples using commercial kits (Illustra Blood genomicPrep Mini Spin kit, GE Healthcare®, Piscataway, NJ). To confirm the presence of reptilian genomic material and ensure sample quality, we used the Polymerase Chain Reaction (PCR) for the reptile cytochrome oxidase subunit I (COI) [21]. Next, we subjected DNA samples to PCR for the partial amplification of the cytochrome b (cyt b) gene, using the oligonucleotides 3760F (5’-GAGTGGATGGTGTTTTAGAT-3’) and 4292R (5’-TGGAACAATATGTARAGGAGT-3’), commonly used for the detection of *Haemoproteus* and *Plasmodium*, amplifying a 533 bp fragment [22].

In the positive samples, we conducted additional molecular tests for blood parasites using Nested PCR, employing the oligonucleotides described by Pacheco and co-workers [23], targeting the mitochondrial gene cytochrome b (*cyt* b, 1,131 bp). In this Nested PCR, we used the external oligonucleotides AE298 (5’-TGTAATGCCTAGACGTATTCC-3’) and AE299 (5’-GTCAAWCAAACATGAATATAGAC-3’), following the same conditions described by Pacheco and co-workers [23], and the internal oligonucleotides AE064 (5’-TCTATTAATTTAGYWAAAGCAC-3’) and AE066 (5-GCTTGGGAGCTGTAATCATAAT-3’). The Nested PCR conditions were as follows: a final volume of 25μL in the reaction with a 10 × buffer solution, 2.0mM MgCl2, 0.2mM dNTP at 25mmol, 0.25μL TaqDNA Polymerase 5U/μL (Invitrogen®, Carlsbad, CA), 1μL of DNA, and 1.0μL of each oligonucleotide (10 mM). The amplification protocol consisted of an initial denaturation at 94 °C for 1 min, followed by 30 cycles of 94 °C for 1 min, 61 °C for 1 min, and 72 °C for 2 mins, with a final extension of 10 mins at 72 °C.

We subjected the PCR products to electrophoresis on a 1.5% (533 bp) and 1.0% (1,131 bp) agarose gel, stained with ethidium bromide, and examined under UV transilluminator light (UV transilluminator®, UVP LLC, Upland, CA). We tested all samples in duplicate (Biorad® C1000TM Thermal Cycler, Hercules, CA). We used ultrapure autoclaved Milli-Q® water as a negative control, while DNA samples from animals naturally infected by haemosporidian parasites were used as positive controls for all testing protocols.

### 2.5 Purification of PCR products, sequencing, and genetic analysis

We purified the amplified products using the PureLink™ Quick Gel Extraction & PCR Purification Combo Kit (Invitrogen®, Carlsbad, CA), according to the manufacturer’s recommendations. Next, we sequenced using the Sanger method on an ABI 3730 XL DNA Analyzer (Applied Biosystems, Foster City, California (CA)), using specific oligonucleotides for each PCR.

We performed sequence trimming and quality analysis in Geneious v. 9.0.5. Then, we produced a consensus sequence for each isolate and evaluated the chromatogram and Phred quality score. We used the BLASTn tool (http://www.ncbi.nlm.nih.gov/BLAST), assessing the sequences from this study against the “non-redundant” (nr) database. We deposited all sequences in the GenBank database (S1 Table).

We used a Bayesian approach to build a phylogenetic tree based on partial fragments of the cytochrome b gene (approximately 500 nt) from 48 *Plasmodium* isolates originating from reptiles in Brazil and other continents. We aligned sequences using the Clustal Omega algorithm and built a phylogenetic tree using MrBayes 3.2.7, employing the General Time Reversible (GTR) model with a gamma distribution (+G) and invariant sites (I+) as the substitution matrix according to the jModelTest v2.1.10 results. We ran the Monte Carlo Markov Chain (MCMC) algorithm for 10,000,000 generations, sampled every 1,000, until convergence, and discarded the first 25% of generations as “burn-in”. We used *Hemoproteus columbae* as an outgroup.

Finally, we used MEGA X to calculate genetic distances (p-distance) for the phylogenetically most similar sequences to the isolates, those with the lowest e-values in BLASTn, and those with a minimum length of 500 nt. We used the Kimura 2-parameter (K2P) model and estimated the alpha parameter beforehand.

## 3. Results

**Fig 1:**
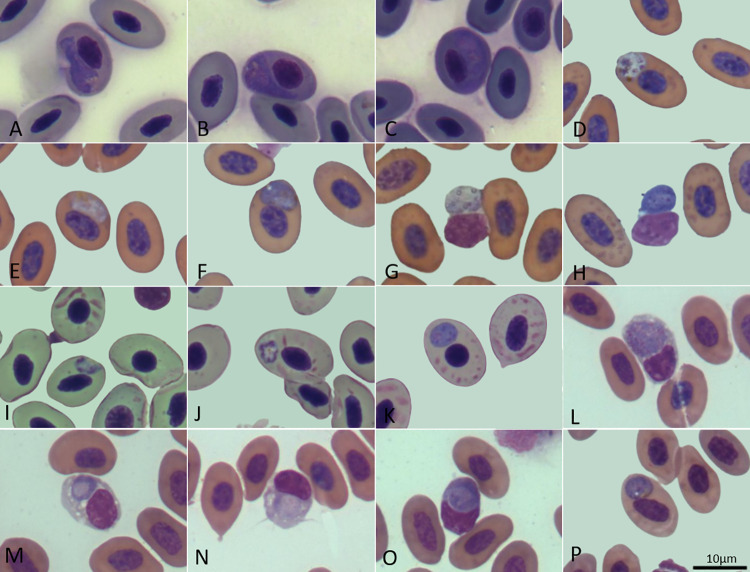
*Plasmodium* sp. 5 infecting *Ameivula ocellifera* (L169), showing meronts (A) and macrogametocytes (B-C). *P. tropiduri* infecting *Tropidurus itambere* (L180) displaying meronts (D), microgametocyte (E), and macrogametocyte (F). We observed co-infection with *P. ouropretensis* in the same lizard, showing microgametocytes (G) and macrogametocytes (H). *Plasmodium* sp. 7 infecting *Ameiva ameiva* (L165), exhibiting meronts (I-J) and macrogametocytes (K). *Plasmodium* sp. infecting *Notomabuya frenata* (L178), presenting forms in non-erythrocytic cells like lymphocytes and thrombocytes (L-O), meronts (L), young gametocytes (M), microgametocytes (N), and macrogametocytes (O). Co-infection with *Plasmodium brasiliensis* which infects erythrocytes (P).

**Fig 2:**
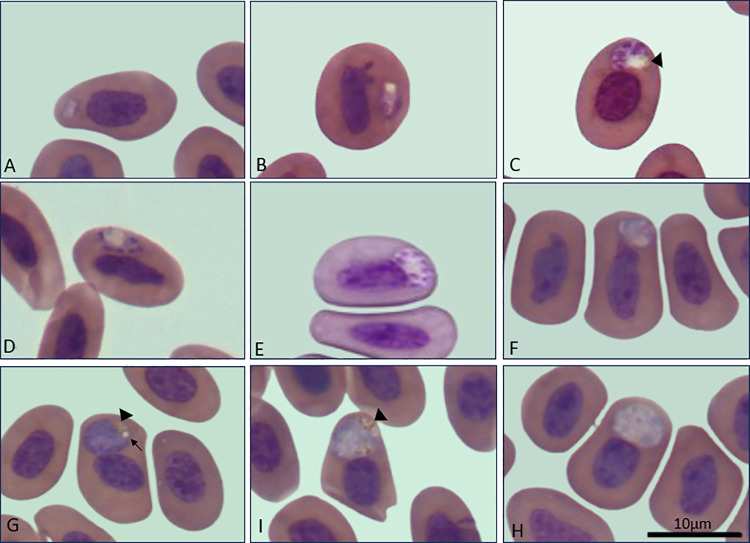
Young trophozoite (A). Mature trophozoite (B). Oval or round-shaped meronts (C, D, E). A rare meront presents more than six nuclei (E). Young gametocyte (F). Macrogametocytes (G). Microgametocytes (H, I). The black head arrow indicates a cluster of malarial pigments found in meronts, with the pigment located inside a large vacuole. The black arrow indicates a small, distinct vacuole present in gametocytes. These images were obtained from individuals L177 and L178.

We collected and analyzed DNA samples from 19 lizard species, totaling 302 samples using PCR. Of these, 61 (20.2%) tested positive for *Plasmodium* spp. (Tables 1 and S2). Intraerythrocytic gametocytes were observed in 18 blood smears. Molecular characterization was further conducted on the 61 positive samples using Nested PCR with specific oligonucleotides. The assay described by Pacheco and co-workers [23] successfully amplified 44 of the 61 samples that were positive in the initial assay described by Beadell and co-workers [22] (Table S2). Among these, 16 samples representing seven species —*Ameiva ameiva, Ameivula ocellifera, Copeoglossum nigropunctatum, Hoplocercus spinosus, Notomabuya frenata, Tropidurus itambere,* and *Tropidurus oreadicus*—were selected for sequencing. These samples were chosen for sequencing based on their origin from different lizard species, their production of amplicons of appropriate size, and their satisfactory concentration. *Plasmodium* spp. were identified both molecularly and morphologically (Fig 1), including the identification of a new species (Fig 2 and Table 2).

**Table 1:** Number of animals per species tested in the molecular screening using the *cytb* 3760F and 4292R oligonucleotides (533 bp).

SPECIES	NUMBER OF POSITIVE ANIMALS	TOTAL	PREVALENCE
*Notomabuya frenata*	12	19	63.16%
*Ameivula ocellifera*	15	29	51.72%
*Tropidurus oreadicus*	18	64	28.13%
*Ameiva ameiva*	7	27	25.93%
*Copeoglossum nigropunctatum*	5	34	14.71%
*Hoplocercus spinosus*	1	9	11.11%
*Brasiliscincus heathi*	2	19	10.53%
*Tropidurus itambere*	1	26	3.85%
*Colobosaura modesta*	0	1	0.00%
*Enyalius capetinga*	0	10	0.00%
*Gymnodactylus*	0	1	0.00%
*Hemidactylus brasilianus*	0	5	0.00%
*Iguana iguana*	0	5	0.00%
*Micrablepharus maximiliani*	0	6	0.00%
*Polychrus acutirotris*	0	1	0.00%
*Salvator merianae*	0	1	0.00%
*Tropidurus semitaeniatus*	0	43	0.00%
*Tropidurus hispidus*	0	1	0.00%
*Tropidurus torquatus*	0	1	0.00%
TOTAL	**61**	**302**	

**Table 2:** Comparison of characteristics of *Plasmodium brasiliensis* n.sp. from this study with *Plasmodium tropiduri* (Lainson and Shaw, 1969) and *Plasmodium tropiduri tropiduri* (Hernandes-Córdoba et al., 2021).

Parasites	*Plasmodium brasiliensis* n.sp.	*Plasmodium tropiduri* Lainson and Shaw (1969)	*Plasmodium tropiduri tropiduri* Hernandes Códoba et al. (2021)
Host cell	Erythrocytes	Erythrocytes	Erythrocytes
Uninfected host cell	n = 10		n = 10
Length	12.7–16.3 (15.1 ± 1.19)		14.7–19 (16.54 ± 1.12)
Width	7.50–9.24 (8.52 ± 0.50)		8.7–11 (9.82 ± 0.78)
Area	75.1–124.0 (104.4 ± 14.71)		
Nucleus length	6.90–8.78 (7.82 ± 0.67)		6.1–8.2 (7.5 ± 0.64)
Nucleus width	2.77–4.37 (7.82 ± 0.52)		4.1–5.1 (4.56–0.32)
Nucleus area	17.5–29.5 (23.8 ± 4.74)		
Trophozoites	n = 10		
Area	3.37–7.11 (4.70 ± 0.12)		
Meronts	n = 10		n = 10
Number of merozoites	4–6 (5 ± 0.84)	6-12	10–25 (15.4 ± 4.45)
Length	3.50–6.06 (4.46 ± 0.68)	5.7	5.6–10.6 (7.5 ± 0.7)
Width	2.70–4.78 (3.76 ± 0.55)		3.7–5.8 (4.9 ± 0.7)
Area	9.32–13.84 (11.12 ± 1.29)		
Length of infected host cell	11.8–17.1 (13.7 ± 1.61)		15–20 (17 ± 1.22)
Width of infected host cell	7.69–9.97 (8.55 ± 0.74)		8–11.2 (10.3 ± 0.9)
Length of infected host cell nucleus	6.00–8.72 (7.45 ± 0.94)		5.7–9 (7.66 ± 1)
Width of infected host cell nucleus	2.92–4.10 (3.55 ± 0.39)		4.8–6.3 (5.23 ± 0.54)
Macrogametocytes	n = 10		
Length	5.27–6.75 (6.08 ± 0.58)	7.5	6.4–10.3 (7.7 ± 0.9)
Width	3.21–5.24 (4.10 ± 0.68)		4.4–6.8 (5.07 ± 0.83)
Area	14.3–25.0 (19.5 ± 4.33)		
Length of infected host cell	11.9–17.1 (14.8 ± 1.75)		15–19 (17.3 ± 1.24)
Width of infected host cell	7.67–9.76 (8.59 ± 0.69)		8.3–10.8 (9.4 ± 0.8)
Length of infected host cell nucleus	6.13–9.04 (7.85 ± 1.00)		3.5–5.1 (7.13 ± 0.8)
Width of infected host cell nucleus	2.77–4.99 (3.70 ± 0.66)		3.8–5.1 (4.1 ± 0.45)
Microgametocytes	n = 10		
Length	4.68–6.59 (5.70 ± 0.60)	7.5	6.4–10.3 (7.7 ± 0.9)
Width	3.97–7.80 (5.35 ± 1.29)		4.4–6.8 (5.07 ± 0.83)
Area	19.0–23.5 (21.7 ± 1.60)		
Length of infected host cell	12.5–15.8 (13.5 ± 1.13)		15–19 (17.3 ± 1.24)
Width of infected host cell	7.71–9.51 (8.40 ± 0.57)		8.3–10.8 (9.4 ± 0.8)
Length of infected host cell nucleus	5.30–7.97 (6.59 ± 0.99)		3.5–5.1 (7.13 ± 0.8)
Width of infected host cell nucleus	3.21–4.66 (3.83 ± 0.52)		3.8–5.1 (4.1 ± 0.45)

Legend: Measurements are in μm and are presented as ranges followed by mean values and standard deviations. Lainson and Shaw [24] and Herdendes Córdoba et al. [8] measured gametocytes, but did not distinguish between microgametocytes and macrogametocytes.

## 4. Description of *Plasmodium (Lacertamoeba) brasiliensis* n. sp.

*Plasmodium brasiliensis* infects the lizard *Notomabuya frenata* (Squamata: Scincidae). This parasite specifically targets mature erythrocytes, with meronts being the most common form observed in blood smears, accounting for 53% of the observed stages. Mature forms, such as meronts and gametocytes, are typically located in the polar or subpolar regions of the infected red blood cells. Some younger forms may be located on the lateral sides of the host cells.

### Description of Fig 2 and Table 2:

Trophozoites: Young trophozoites have a droplet-shaped morphology (Fig 2A). As the parasite matures, it develops a vacuole (Fig 2B), containing a yellowish malarial pigment mass.

Meronts: Meronts typically contain 4 to 6 nuclei and feature a large vacuole with a yellowish mass of pigments, usually located on one edge of the parasite. The shape of meronts can vary from round to oval (Figs 2C, 2D). These parasites are generally found near or attached to the host cell’s nucleus. Meronts with more than six nuclei (specifically 7 to 8) are rare (Fig 2E); only three meronts out of 60 evaluated present more than six nuclei.

Gametocytes: Gametocytes are similar to *P. tropiduri*, presenting a rounded or oval shape. They are typically found in the polar or subpolar region of the infected red blood cells (Figs 2F–2I). The sexual forms are roughly the same size as the host cell’s nucleus, and they either displace or adhere to it. The young gametocytes have a yellowish malarial pigment area (Fig 2F). Macrogametocytes (Fig 2G) and microgametocytes (Figs 2H, 2I) display distinct characteristics in their blue-stained cytoplasm and chromatin condensation, while both maintain a consistent size of approximately 20 µm in the parasite area. Additionally, this parasite contains noticeable vacuoles, observed in five out of ten macrogametocytes and four out of eight microgametocytes. The yellowish malaria pigment is usually found clustered at the edge of the parasite, replacing the vacuoles present in other stages, such as meronts and trophozoites.

*Type Host: Notomabuya frenata* (Scincidae).

*Type Locality:* IBGE Ecological Reserve, Brasília, Distrito Federal, Brazil (47°53’07” W, 15°56’41” S).

*Distribution:* Only known from type-locality.

*Type specimen:* Hapantotype L177. Intensity of infection was 0.077. Collected by Izabelle T. S. Carvalho and Adriana P. Furtado and deposited in the Institute of Biological Sciences (Universidade Federal de Minas Gerais, Belo Horizonte, Brazil).

*DNA Sequences:* PP489230, PP489232, PP489233, PP489234

*Other Hosts:* Unkwown

*Other Localities:* Unkwown.

*ZooBank registration*: The Life Science Identifier (LSID) of the article is urn:lsid:zoobank.org:pub:26592D81-D9F4-4BB3-88D1-02CFDB381AD9. The LSID for the new name *Plasmodium brasiliensis* n. sp. is urn:lsid:zoobank.org:act:25FD6C39-0ECD-41A0-9D43-5C3AD31DDEAE.

*Etymology*: *P. brasiliensis* name is derived from the name of Brasília city, located in the Federal District, the capital of Brazil, where the infected lizard was sampled.

*Remarks:* In 1969, Lainson and Shaw [24] described the species *Plasmodium tropiduri* in the lizard *Mabuya mabouya*. In this host, *P. tropiduri* produced 4 to 6 nuclei, contrasting observations in tropidurids, where between 4 and 26 nuclei were recorded. Subsequently, Telford [25] proposed the use of genomic data to compare *P. tropiduri* found in skinks with those found in tropidurids. This comparison aims to determine whether they represent the same species and can infect two distinct families of reptiles. Our findings indicate that *P. tropiduri* from tropidurids differs from the newly identified *P. brasiliensis* based on differences in the number of meront nuclei and a 4.1% sequence divergence in the cytb gene, suggesting significant divergence between these two parasites.

**Fig 3:**
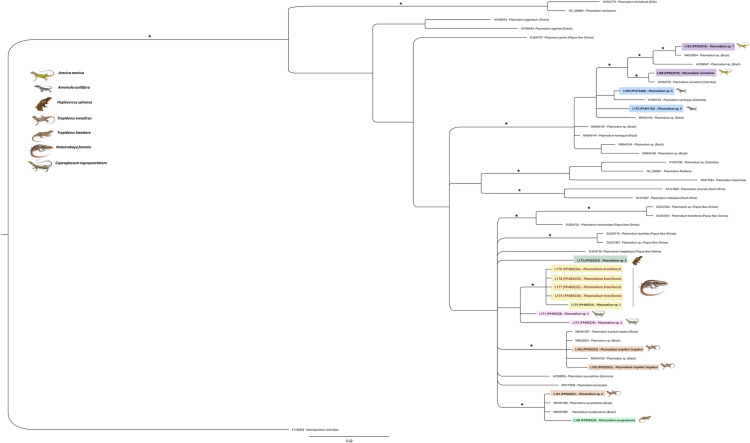
Phylogenetic tree generated using Bayesian analysis of partial *cytb* gene fragments ( **~500 nt) from 48** ***Plasmodium*** **isolates.** Posterior probabilities of 0.95 or higher are indicated with asterisks. Sequences obtained in this study are highlighted in colors corresponding to the identified host species. The scale bar represents the number of substitutions per site. *Hemoproteus columbae* was used as the outgroup.

The sequences from *Notomabuya frenata* (L174, L178, L177, L179, and L175), collected in the Distrito Federal region, formed a well-supported monophyletic clade, phylogenetically distinct from previously described *Plasmodium* species (Fig 3). The sequences from *Copeoglossum nigropunctatum* (L171 and L172), also from the Distrito Federal, grouped with the sequences from *N. frenata* (L174, L178, L177, L179, and L175); however, this grouping exhibited low support. The sequences from *N. frenata* (L174, L178, L177, L179, and L175) exhibited 100% identity among themselves, except for sequence L175, which differed by only two nucleotides, showing 99.7% identity. The sequences from *C. nigropunctatum* (L171 and L172) showed 98.2% identity and a genetic distance of 1.8% between them (Table 3). When comparing the identity of sequences L171 and L172 with L174, L177, L178, and L179, the identities obtained were 98.9% and 98%, respectively. For isolate L175, the identities were 98.6% and 97.7%, respectively.

**Table 3:** Pairwise genetic distance matrix based on the mitochondrial *cytb* gene among *Plasmodium* sequences from lizards.

	L181	MW491390	MW491389	L180	L165	L168	KY653755	L169	KY653753	L170	MN540144	L173	L179	L178	L177	L174	L175	L171	L172	MW491387	L186	L185
**L181 (PP502921)**																						
*P. ouropretensis* (MW491390)	0.0%																					
*P. ouropretensis* (MW491389)	0.0%	0.0%																				
**L180 (PP502920)**	0.7%	0.7%	0.7%																			
**L165 (PP502918)**	6.4%	6.4%	6.4%	6.7%																		
**L168 (PP502919)**	6.0%	6.0%	6.0%	6.2%	1.4%																	
*P. carmelinoi* (KY653755)	6.0%	6.0%	6.0%	6.2%	1.4%	0.0%																
**L169 (PP472489)**	5.3%	5.3%	5.3%	5.5%	2.1%	1.6%	1.6%															
*P. kentropyxi* (KY653753)	6.0%	6.0%	6.0%	6.2%	2.8%	2.3%	2.3%	0.7%														
**L170 (PP481182)**	5.3%	5.3%	5.3%	5.1%	2.1%	1.6%	1.6%	0.9%	1.6%													
*P. kentropyxi* (MN540144)	5.3%	5.3%	5.3%	5.5%	2.1%	1.8%	1.8%	1.1%	1.8%	1.6%												
**L173 (PP502924)**	4.1%	4.1%	4.1%	3.9%	5.5%	5.1%	5.1%	4.8%	5.1%	4.4%	4.4%											
**L179 (PP489234)**	3.0%	3.0%	3.0%	3.2%	6.2%	5.7%	5.7%	5.1%	5.7%	5.1%	4.6%	3.0%										
**L178 (PP489233)**	3.0%	3.0%	3.0%	3.2%	6.2%	5.7%	5.7%	5.1%	5.7%	5.1%	4.6%	3.0%	0.0%									
**L177 (PP489232)**	3.0%	3.0%	3.0%	3.2%	6.2%	5.7%	5.7%	5.1%	5.7%	5.1%	4.6%	3.0%	0.0%	0.0%								
**L174 (PP489230)**	3.0%	3.0%	3.0%	3.2%	6.2%	5.7%	5.7%	5.1%	5.7%	5.1%	4.6%	3.0%	0.0%	0.0%	0.0%							
**L175 (PP489231)**	3.4%	3.4%	3.4%	3.7%	6.2%	5.7%	5.7%	5.1%	5.7%	5.5%	4.6%	3.0%	0.5%	0.5%	0.5%	0.5%						
**L171 (PP489228)**	2.5%	2.5%	2.5%	2.8%	5.7%	5.3%	5.3%	4.6%	5.3%	4.6%	4.1%	2.5%	0.9%	0.9%	0.9%	0.9%	1.4%					
**L172 (PP489229)**	2.5%	2.5%	2.5%	2.8%	6.2%	5.7%	5.7%	5.1%	5.7%	5.1%	5.1%	3.4%	1.8%	1.8%	1.8%	1.8%	2.3%	1.8%				
*P. t. tropiduri* (MW491387)	3.0%	3.0%	3.0%	3.2%	5.7%	5.3%	5.3%	4.1%	4.8%	5.1%	5.1%	3.9%	4.1%	4.1%	4.1%	4.1%	4.1%	3.7%	3.7%			
**L186 (PP502923)**	3.0%	3.0%	3.0%	3.2%	5.7%	5.3%	5.3%	4.1%	4.8%	5.1%	5.1%	3.9%	4.1%	4.1%	4.1%	4.1%	4.1%	3.7%	3.7%	0.0%		
**L185 (PP502922)**	3.4%	3.4%	3.4%	3.7%	5.5%	5.1%	5.1%	3.9%	4.6%	4.8%	4.8%	3.7%	3.9%	3.9%	3.9%	3.9%	3.9%	3.4%	3.7%	0.5%	0.5%	
*P. azurophilum* (AY099055)	2.5%	2.5%	2.5%	2.8%	6.5%	6.0%	6.0%	5.3%	6.0%	5.8%	5.1%	3.0%	3.2%	3.2%	3.2%	3.2%	3.2%	2.8%	3.2%	2.8%	2.8%	2.8%

In addition to the infection observed in erythrocytes in the blood smear, sample L178 also presented coinfection by *Plasmodium* sp., with parasites infecting non-erythrocytic cells as well (Figs 1L–1O).

Lizards in the genus *Tropidurus* were parasitized by two isolates (L180 and L181). In BLASTn, sequence L180 from a *T. itambere* lizard from Distrito Federal showed 99.36% identity with 100% coverage to isolate MW491390 of *Plasmodium ouropretensis* deposited in GenBank (Table 4). Conversely, sample L181 collected in Piauí from *T. oreadicus* displayed 100% identity with 100% coverage to isolate MW491389 of *P. ouropretensis* in GenBank (Table 4). In the phylogenetic tree, these sequences clustered with strong support (Fig 3). The identity observed between isolates L180 and L181 was 99.4%, differing by three nucleotides. Both samples exhibited blood smears containing structures indicative of haemosporidian parasites, however, only L180 achieved sufficient quality for morphological analysis, showing co-infection of *P. tropiduri* and *P. ouropretensis* (Figs 1D–1H).

**Table 4:** Identity values obtained through BLASTn analysis of the tested isolates (E-value =  0.0).

SEQUENCES	SEQUENCE SIZE (BP)	IDENTITY (%)	COVERAGE (%)	SEQUENCES WITH HIGHEST IDENTITY	COUNTRY	HOST
**L165** *A. ameiva*	559	100%	100%	***Plasmodium* sp**. isolate TEC6560 cytochrome b (*cytb*) gene, partial cds; mitochondrial. MK033604.1	Brazil	*A. ameiva*
**L168** *A. ameiva*	834	99.88%	100%	***Plasmodium carmelinoi*** clone GU040C cytochrome b (*cytb*) gene, partial cds; mitochondrial KY653755.1	Colombia	*A. ameiva*
**L169** *A. ocellifera*	823	99.4%	100%	***Plasmodium kentropyxi*** clone GU027A cytochrome b (*cytb*) gene, partial cds; mitochondrial. KY653753.1	Colombia	*Cnemidophorus* cf*. gramivagus*
**L170** *A. ocellifera*	831	98.19%	100%	***Plasmodium kentropyxi*** clone GU027A cytochrome b (*cytb*) gene, partial cds; mitochondrial. KY653753.1	Colombia	*Cnemidophorus* cf*. gramivagus*
**L171** *C. nigropunctatum*	788	98.22%	100%	***Plasmodium ouropretensis*** isolate UFMG37 clone UFMG37DA mitochondrion, complete genome MW491389.1	Brazil	*Tropidurus torquatus*
**L172** *C. nigropunctatum*	582	97.93%	99%	***Plasmodium ouropretensis*** isolate UFMG123 clone UFMG123DAA mitochondrion, complete genome. MW491390.1	Brazil	*Tropidurus torquatus*
**L173** *H. spinosus*	907	98%	100%	***Plasmodium azurophilum*** from *Anolis oculatus* cytochrome b gene, partial cds; mitochondrial gene for mitochondrial product. AY099055.1	Dominican Republic	*Anolis oculatus*
**L174** *N. frenata*	672	97.47%	100%	***Plasmodium ouropretensis*** isolate UFMG37 clone UFMG37DA mitochondrion, complete genome. MW491389.1	Brazil	*Tropidurus torquatus*
**L175** *N. frenata*	672	97.17%	100%	***Plasmodium ouropretensis*** isolate UFMG37 clone UFMG37DA mitochondrion, complete genome. MW491389.1	Brazil	*Tropidurus torquatus*
**L177** *N. frenata*	908	97.80%	100%	***Plasmodium ouropretensis*** isolate UFMG37 clone UFMG37DA mitochondrion, complete genome. MW491389.1	Brazil	*Tropidurus torquatus*
**L178** *N. frenata*	788	97.59%	99%	***Plasmodium ouropretensis*** isolate UFMG37 clone UFMG37DA mitochondrion, complete genome. MW491389.1	Brazil	*Tropidurus torquatus*
**L179** *N. frenata*	873	97.71%	100%	***Plasmodium ouropretensis*** isolate UFMG37 clone UFMG37DA mitochondrion, complete genome. MW491389.1	Brazil	*Tropidurus torquatus*
**L180** *T. itambere*	621	99.36%	100%	***Plasmodium ouropretensis*** isolate UFMG123 clone UFMG123DAA mitochondrion, complete genome. MW491390.1	Brazil	*Tropidurus torquatus*
**L181** *T. oreadicus*	637	100%	100%	***Plasmodium ouropretensis*** isolate UFMG37 clone UFMG37DA mitochondrion, complete genome. MW491389.1	Brazil	*Tropidurus torquatus*
**L185** *T. oreadicus*	919	99.78%	100%	***Plasmodium tropiduri*** tropiduri isolate UFMG29 clone UFMG29A mitochondrion, complete genome. MW491387.1	Brazil	*Tropidurus torquatus*
**L186** *T. oreadicus*	869	100%	100%	***Plasmodium sp.*** isolate TEC4774 cytochrome b (*cytb*) gene, partial cds; mitochondrial. MK033603.1	Brazil	*Tropidurus hispidus*

Sequences L185 and L186, derived from *Tropidurus oreadicus* lizards in Piauí, exhibited 99.7% identity between themselves. Upon analyzing the identity values obtained from BLASTn (Table 4), it was observed that sequence L185 displayed 99.78% identity with 100% coverage to isolate MW491387 of *Plasmodium tropiduri tropiduri*, identified in a *T. torquatus* individual in Brazil. Conversely, sequence L186 showed 100% identity with 100% coverage to sequence MK033603 of *Plasmodium* sp. in a *T. hispidus* individual from Brazil. In the phylogenetic tree, both sequences clustered with lineages containing *Plasmodium* sp. isolates (MK033603 and MN540150) and *P. tropiduri tropiduri* (MW491387) identified in Brazil (Fig 3).

Two sequences from *Ameivula ocellifera* lizards (L169 and L170) collected in Piauí exhibited a similarity of 98.8%. Sequence L169 exhibited 99.4% identity with sequence KY653753 of *Plasmodium kentropyxi*, obtained in Colombia from a lizard (*Cnemidophorus cf. gramivagus*) (Table 4). The two sequences differ by only five nucleotides. In the phylogenetic analysis, L169 and KY653753 formed a monophyletic clade; however, this grouping was weakly supported (Fig 3). In the morphological analysis, infected meronts and macrogametocytes were observed (Figs 1A–1C).

Sequence L170 (from *A. ocellifera*) displayed 98.19% identity with *P. kentropyxi* (KY653753). Although phylogenetically close to KY653753 and L169, it failed to cluster within a strongly supported clade in the phylogenetic analysis (Fig 3). The *Ameiva ameiva* isolates collected in Piauí (L165) and Distrito Federal (L168) exhibited a 98.6% sequence identity with each other. Isolate L165 displayed 100% identity with 100% coverage to *Plasmodium* sp. (MK033604) derived from *Ameiva ameiva*, which was also observed in our isolate (see Table 4). L165 clustered with MK033604 and AY099047, forming a well-supported group. In the morphological analysis, infected meronts and macrogametocytes were observed (Figs 1I–1K). Sequence L168, obtained from *Ameiva ameiva* in the Federal District, exhibited 99.88% identity and 100% coverage with sequence KY653755 of *Plasmodium carmelinoi*, identified in a conspecific lizard from Colombia (see Table 4). In the phylogenetic tree, both sequences formed a strongly supported monophyletic group (see Fig 3).

The isolate L173, obtained from *Hoplocercus spinosus* in Piauí, exhibited a 98% sequence identity with 100% coverage to the sequence AY099055 of *Plasmodium azurophilum* found in *Anolis oculatus* in Dominica. The genetic distance between them was 3.0%, based on an alignment of 907 base pairs (bp).

## 5. Discussion

The 100% sequence identities observed among the four samples (L174, L177, L178, and L179), combined with the genetic distances relative to other *Plasmodium* species described in lizards (Table 3), provide strong evidence for their classification as a single *Plasmodium* species. The clustering of these sequences into a well-supported monophyletic clade, alongside the formation of an independent lineage (Fig 3), further substantiates this classification. Based on these findings, we propose the new species *Plasmodium brasiliensis* n. sp. While morphological analysis revealed some similarities with *P. tropiduri*, notable differences, such as the number of nuclei in meronts and genetic divergence, clearly distinguish the two species (Fig 2 and Table 2). Furthermore, co-infection with *Plasmodium brasiliensis* and *Plasmodium* sp. was observed in sample L178, with the latter infecting non-erythrocytic cells (Figs 1L–1O).

Although sequence L175 clusters within a well-supported monophyletic clade with sequences L179, L178, L177, and L174, its genetic distance of 0.5% and the lack of morphological characterization of this isolate prevent L175 (*Plasmodium* sp. 1) from being designated as *Plasmodium brasiliensis*.

On the other hand, sequences L171 and L172, obtained from *Copeoglossum nigropunctatum*, clustered with the sequences of *P. brasiliensis* but exhibited low phylogenetic support and genetic distances of 0.9% and 1.8% relative to the hapantotype. These differences, combined with their association with a distinct host species, supported their provisional classification as *Plasmodium* sp. 2 and *Plasmodium* sp. 3, respectively.

The findings highlight the discovery of a new *Plasmodium* species in the Federal District, proposed as *Plasmodium brasiliensis* n. sp., based on four isolates obtained from a previously unexplored region for these parasites. These results underscore the importance of broad and comprehensive sampling efforts in studies of hemoparasites in reptiles. Limited sampling can lead to underrepresentation of host taxa, compromising accurate species classification and hindering the understanding of genus-level subdivisions [9]. This study represents the first and most extensive molecular survey of hemosporidian agents infecting a diverse array of free-ranging lizards in the Brazilian Cerrado, providing significant contributions to the understanding of the diversity and distribution of these parasites.

The BLASTn identity scores and the formation of a well-supported monophyletic clade in the phylogenetic tree, involving the *Tropidurus* isolates L180 and L181 (Fig 3) and the *Plasmodium ouropretensis* sequences available in GenBank (Table 4), provide strong evidence of the presence of two new isolates of *P. ouropretensis* in Brazil, specifically in the Federal District and Piauí regions. Morphological analysis of the blood smears corroborated the molecular data for isolate L180, confirming the infection by *P. ouropretensis*. However, for isolate L181, the poor quality of the slides precluded a definitive morphological identification, leading to a provisional classification as *Plasmodium* sp. 4, despite its consistent positioning within the *P. ouropretensis* clade in the phylogenetic tree. These findings extend the known geographic distribution of this parasite in lizards, which had previously been reported only in *T. torquatus* in Southeast Brazil [8]. This species was recently described by Córdoba and co-workers [8], who identified two distinct phylogenetic lineages of samples morphologically similar to *P. tropiduri tropiduri*, one infecting erythrocytes and the other infecting non-erythrocytic cells. The lineage found in erythrocytes shared a most recent common ancestor with *Plasmodium* infecting erythrocytes detected in Colombia (*P. carmelinoi* and *P. kentropyxi*) [see 26] and was designated as *P. tropiduri tropiduri*, providing the first report with morphological and molecular data for this species [see 8]. In the phylogenetic tree, the non-erythrocytic lineage did not form a monophyletic clade with *Plasmodium leucocytica*, a parasite that infects white blood cells in Caribbean lizards [8,27], in contrast to other previously described phylogenetic trees [8]. This difference may be attributed to the use of short fragments and different genetic regions in the alignment.

Samples L180 and L181 exhibited erythrocytes infected with structures suggestive of hemosporidian parasites in blood smears. Morphological analysis confirmed the presence of co-infection with *Plasmodium tropiduri* in sample L180 (Figs 1D–1F). According to Córdoba et al. [8], *Plasmodium t. tropiduri* and *P. ouropretensis* are morphologically similar, and despite limited data, *P. ouropretensis* has frequently been observed in sympatry with populations of *P. t. tropiduri*. In addition to the study conducted in Minas Gerais, co-infections between these species have also been reported in São Paulo [28] and Guárico, Venezuela [29]. These findings support our results and extend the knowledge on the distribution of these mixed infections within Brazil, now including the Federal District (this study). Such co-occurrences may suggest an ecological interaction between the species, possibly facilitating the persistence and dispersion of these parasitic lineages.

For sample L181, it is plausible that the same co-infection principle observed in sample L180 also occurred, potentially involving *Plasmodium* spp. in *Tropidurus oreadicus* from the state of Piauí, Brazil. However, the poor quality of the slides from this sample prevented the definitive identification of the co-infection. The lack of more robust data for sample L181 limits the confirmation of the presence of *P. tropiduri* or *P. ouropretensis* in this location and host species, underscoring the need for further analyses to validate these findings and enhance our understanding of the distribution and dynamics of mixed infections among different lizard species in Brazil.

Based on the identity scores ranging from 99.78% to 100% and the phylogenetic analysis forming a well-supported monophyletic group, it was possible to attribute sequences L185 and L186 as new isolates of *Plasmodium t. tropiduri*. We also included in this classification the isolates previously described as *Plasmodium* sp., the sequences deposited as MK033603 in *Hemidactylus mabouia* [12], and MN540150 in *Strobilurus torquatus* [3], all from northeastern Brazil. These sequences exhibited 99.9% and 99.6% identity, respectively, with *P. t. tropiduri* [8].

The results obtained from sequences L169 and L170 reveal two additional *Plasmodium* sp. lineages (*Plasmodium* sp. 5 and 6, respectively) in Brazil, marking the first report of *Plasmodium* infection in the lizard species *Ameivula ocellifera*. Both isolates are from the same geographic region, Piauí, Brazil, and cluster with the *P. kentropyxi* lineage from Colombia [26], although with weak support. Morphological identification of these lineages was not possible due to the insufficient number of observed morphological forms; only forms within proerythrocytes were identified in the L169 isolate. However, it is noteworthy that the intraspecific diversity of *P. kentropyxi* is remarkable. To date, none of the sequences described for this species in lizards [3,26] exhibited 100% identity among themselves, with an overall identity of 99.1% between isolates. Additional sequences and morphological data are needed for a more comprehensive characterization of the genetic diversity of this species.

The L168 sequence, with 99.88% identity, consistently clusters with the isolate identified in Colombia [26], supporting its classification as *Plasmodium carmelinoi*, now reported in the Federal District, Brazil. In contrast, the L165 (*Plasmodium sp.* 7) sequence forms a distinct clade alongside isolates MK033604 [12] and AY099047 [2], classified as *Plasmodium sp.* Collectively, these sequences constitute a larger group that is subdivided into two well-supported monophyletic clades.

*Plasmodium azurophilum* was first described by Telford in 1975 [30] as a species with two genetically distinct lineages: one infecting erythrocytes and the other infecting leukocytes. The leukocytic lineage, initially hypothesized to be derived from the erythrocytic lineage and altering its life history to infect white blood cells, was later designated *Plasmodium leucocytica* by Telford [27]. However, this hypothesis was proven incorrect following sequencing data provided by Perkins [6], which demonstrated the independent origins of these lineages. To date, *P. azurophilum* has been identified in *Anolis* species in the Caribbean [2,27,31]. The L173 isolate (*Plasmodium* sp. 8) analyzed in this study exhibited 98% identity with 100% coverage and a genetic distance of 3.0% compared to the erythrocytic lineage of *Plasmodium azurophilum* (AY099055) previously identified in *Anolis oculatus* from Dominica [2]. However, these isolates did not form a monophyletic clade, likely due to the limited phylogenetic resolution provided by the fragment of the cytochrome b gene analyzed in this study. This underscores the importance of incorporating additional molecular markers or more informative genomic regions to enhance phylogenetic resolution and strengthen evolutionary inferences for *Plasmodium* species in lizards. Furthermore, the combined morphological and molecular data do not provide sufficient evidence to definitively confirm the existence of a new lineage of *P. azurophilum* in Brazil. Nonetheless, this study constitutes the first record of infection by organisms of the phylum Apicomplexa in *H. spinosus* lizards, significantly contributing to the understanding of parasitic diversity within this host group.

## 6.Conclusion

The extensive diversity of *Plasmodium* species found in lizards within the Cerrado biome and transitional areas is remarkable. In this study, we conducted the first comprehensive survey of *Plasmodium* infections in free-living lizards from the Brazilian Cerrado, employing molecular techniques and microscopic analyses. We described new isolates of *P. ouropretensis*, *P. t. tropiduri*, and *P. carmelinoi*. Furthermore, we identified a novel species, proposed as *Plasmodium brasiliensis* n. sp., through robust analysis encompassing fuor isolates from the host species *N. frenata*.

Our research expands the known global distribution of these species and their host associations, including the previously unreported presence of *Plasmodium sp.* in *H. spinosus* from Brazil. This study also highlights the critical importance of ongoing research in addressing current knowledge gaps and generating new insights into hemosporidian parasites in lizards, as well as the biodiversity hotspot represented by the Cerrado biome.

## Supporting information

S1 Table GenBank Accession Codes for *Plasmodium* Species Identified in This Study. Legend: In the “Sampling Location” column, entries marked with an asterisk (*) correspond to samples collected from Serra das Confusões National Park, Redenção do Gurguéia, Piauí, Brazil (coordinates: 9°28’1.11” S, 44°25’20.31” W; 44°21’33.91” W; 9°25’20.17” S; 44°19’56.14” W; 9°28’26.74” S; 44°18’27.14” W; 9°27’47.49” S). Entries marked with a bullet point (•) correspond to samples collected from IBGE Ecological Reserve, Brasília, Federal District, Brazil (coordinates: 15°56’41” S, 47°53’07” W). (XLSX)

S2 Table List of lizard species samples that tested positive for Haemosporida in molecular (PCR) and microscopic analysis, categorized by the collection region and sex. Legend: PI: Piauí; MT: Mato Grosso; DF: Federal District; M: Male; F: Female; NI: Non-identified; * (Sequences from this study). (XLSX)

S3 Table Matrix of Pairwise Genetic Distances Based on the Mitochondrial *cytb* Gene Using the Phylogenetically Closest Sequences to the isolates. (XLSX)
